# Pediatric HIV care and treatment services in Tanzania: implications for survival

**DOI:** 10.1186/s12913-017-2492-9

**Published:** 2017-08-07

**Authors:** G Somi, M Majigo, J Manyahi, J Nondi, J Agricola, V Sambu, J Todd, A Rwebembera, N Makyao, A Ramadhani, MIN Matee

**Affiliations:** 1 National AIDS Control Programme, Ministry of Health, Community Development, Gender, Elderly and Children, Mwanza, Tanzania; 2 Department of Microbiology and Immunology, School of Medicine, Muhimbili University of Health and Allied Sciences, Mwanza, Tanzania; 3 London School of Hygiene and Tropical Medicine and National Institute for Medical Research (NIMR), Mwanza, Tanzania

**Keywords:** Children, HIV, Enrolment, ART, Care, Mortality, Survival

## Abstract

**Background:**

Improving child survival for HIV-infected children remains an important health agenda. We present progress regarding care and treatment services to HIV infected children in Tanzania.

**Methods:**

The National AIDS Control Programme Care and Treatment (CTC 2) database was used to obtain information of all children aged 0-14yearsenrolled in the HIV Care and Treatment Program between January 2011 and December 2014. We assessed eligibility for ART, time from enrolment to ART initiation, nutritional status, and mortality using Kaplan-Meier methods.

**Results:**

A total of 29,531 (14,304 boys and 15,227 girls) ART-naive children aged 0–14 years were enrolled during the period, approximately 6700 to 8000 children per year. The male to female ratio was 48:50. At enrolment 72% were eligible for ART, 2–3% of children were positive for TB, and 2–4% were severely malnourished.

Between 2011 and 2014, 2368 (8%) died, 9243 (31%) were Lost to Follow-up and 17,920 (61%) were on care or ART. The probability of death was 31% (95% CI 26–35), 43% (40–47), 52% (49–55) and 61% (58–64) by 1,2, 5 and 10 years of age, respectively. The hazard of death was greatest at very young ages (<2 years old), and decreased sharply by 4 years old. Children who were on ART had around 10–15% higher survival over time.

**Conclusions:**

Significant progress has been made regarding provision of paediatric HIV care and treatment in Tanzania. On average 7000 children are enrolled annually, and that approximately two thirds of children diagnosed under the age of 2 years were initiated on ART within a month. Provision of ART as soon as the child is diagnosed is the biggest factor in improving survival. However we noted that i) most children had advanced disease at the time of enrolment ii) approximately two-thirds of children were missing a baseline CD4 measurement and only 35% of children had either a CD4 count or percentage recorded, indicating limited access to CD4 testing services, and iii) 31% were lost to follow-up (LTFU).

These challenges need to be addressed to improve early detection, enrolment and retention of HIV-infected children into care and improve documentation of services offered.

## Background

The 2015 Spectrum model estimates 142,000 Tanzanian children, under the age of 15 years, are infected with human immunodeficiency virus (HIV) [1], and current guidelines state all should be given Antiretroviral Therapy (ART) [2]. Without ART approximately 50% of HIV infected children die before the age of two, and one-third of those who survive past two die before 5 years of age [3]. Improving child survival for HIV exposed and infected children remains an important agenda in Tanzania [4]. Promoting universal access to available HIV care and treatment services is important for improving survival of HIV-infected individuals, especially children [5].

The encouraging success of ART, which includes effective prevention of mother-to-child transmission, has led to ambitious calls to eliminate HIV as a public health threat by 2030 [6]. Achievement of these goals, including the United Nations Programme on HIV/AIDS (UNAIDS) 90–90-90 targets, which aim to have 90% of people living with HIV know their status, 90% of those detected treated with ART, and 90% of those receiving treatment achieving viral load suppression [7], requires a coordinated global scale-up of prevention programmes, pre-exposure prophylaxis (PrEP), and detection and treatment programmes [8].

On its part, the government of the United Republic of Tanzania has laid out goals aiming at zero new HIV infections, zero discrimination and zero HIV related deaths in the population, including children, by 2017 [9]. These goals guide interventions that contribute to improving access to HIV services, especially for vulnerable groups like children.

It is well known that universal access to HIV care and treatment services contributes to improved quality of life, and thus economic, social and political development of society [10], which is in keeping with the United Nations (UN) sustainable goals number 3 [11]. The National AIDS Control Programme (NACP) in Tanzania is aiming at providing antiretroviral medicines (ARVs) to People Living with HIV and AIDS (PLHIV). The main focus is to improve access to ART services in order to reduce HIV related morbidity and mortality, especially among children. Due to this, Ministry of Health in Tanzania requires that paediatric HIV statistics be made available and utilized for policy and decision-making. This report summarizes progress made in providing HIV care and treatment services to children in Tanzania and highlights challenges in achieving universal access for ART to HIV-infected children. The information in this report contributes to the monitoring of Pediatric HIV and AIDS program in Tanzania.

## Methods

### Design, setting and population

This was a descriptive analytical study based on NACP data of children aged 0–14 that was routinely collected from 740 HIV care and treatment service delivery clinics between 2011 and 2014 and archived in the NACP database, where data was extracted.

### Data management and analysis

Primary data generated in the HIV care and treatment clinics (CTC) was captured on facility-held information collection tools designated as CTC-2 card. This was submitted on quarterly basis to national level at NACP in either paper form or electronic system, and subsequently entered in the national care and treatment database designated as CTC-3. The CTC-3 database contains both patient-level section (CTC-3 Macro), exported electronically from clinics using an electronic CTC-2 database and aggregate-level section (CTC-3) that contains data receive quarterly summary reports either paper based or extracted from the CTC-2 data. Data analysis was performed using Stata version 14 [12].

### Eligibility for ART

We determined eligibility of children for ART based on WHO staging or CD4 results and estimated the time from enrolment to ART initiation. Results for eligibility for ART are restricted to 2012 onwards, since the national guidelines changed in 2012, which advocate initiation of ART regardless of World Health Organization (WHO) clinical stage and CD4 cell count [13]. The previous eligibility criteria, before the 2012 guidelines, was CD4 < 25% or WHO stage 3 and 4. A window of −90 to +30 days relative to the date of ART start was allowed to capture characteristics at ART start choosing that closest measurement to ART start, with preference for before.

### Assessment of nutritional status

Nutritional status of children was assessed over time using weight and length or height for age. The nutrition status was translated using Z-score criteria of Weight for age and weight for length/Height. Time was categorized into 3 monthly periods and the highest non-missing nutritional status for each child in each 3 months period was used.

### Estimation of mortality

Mortality was estimated using Kaplan-Meier methods from date of birth, with entry into the risk set from the time of enrolment through death. Lost to follow up (LTFU) was defined if a child was not seen within the 6 months before the end of December 2014 and censored at their last visit date. Children who had not died and were not defined as LTFU were considered to be in follow up through to the analysis censoring date of 31 December 2014 and were censored at that time. Children were defined as No Longer On Treatment (NLOT) if they had either died or were LTFU.

### Ethical consideration and approval

Data used in this analysis was collected routinely by health facilities providing HIV care and treatment services following the National Guidelines for HIV Care and Treatment, in which ethical issues are strongly advocated. Permission to analyse and publish this data was granted by the Ministry of Health, Community Development, Gender, Elderly and Children (MoHCDGEC). Management of the data was under strict supervision the Epidemiology Unit of NACP.

## Results

### Children characteristics at CTC enrolment

A total of 29,531 (14,304 (48%) boys and 15,227 (52%) girls) ART-naive children aged 0–14 years were enrolled between 2011 and 2014, which is approximately 6700 to 8000 children per year. The characteristics of enrolees are shown in Table 1.

**Table 1:** Baseline characteristics of 29,531 children enrolled into HIV care services between 2011 and 2014

		2011	2012	2013	2014	Total
Total (row %)		8028 (27.2%)	6727 (22.8%)	7171 (24.3%)	7605 (25.8%)	29,531 (100%)
Sex	Male	3913 (48.7%)	3262 (48.5%)	3418 (47.7%)	3711 (48.8%)	14,304 (48.4%)
	Female	4115 (51.3%)	3465 (51.5%)	3753 (52.3%)	3894 (51.2%)	15,227 (51.6%)
WHO stage	Stage I	2083 (25.9%)	1732 (25.7%)	1685 (23.5%)	1713 (22.5%)	7213 (24.4%)
	Stage II	2417 (30.1%)	1838 (27.3%)	1934 (27.0%)	2045 (26.9%)	8234 (27.9%)
	Stage III	2420 (30.1%)	2240 (33.3%)	2686 (37.5%)	3044 (40.0%)	10,390 (35.2%)
	Stage IV	917 (11.4%)	805 (12.0%)	768 (10.7%)	706 (9.3%)	3196 (10.8%)
	Missing	191 (2.4%)	112 (1.7%)	98 (1.4%)	97 (1.3%)	498 (1.7%)
Weight, kg	<10	2121 (26.4%)	1850 (27.5%)	1845 (25.7%)	1850 (24.3%)	7666 (26.0%)
	10- < 20	3129 (39.0%)	2542 (37.8%)	2855 (39.8%)	3003 (39.5%)	11,529 (39.0%)
	20- < 30	1582 (19.7%)	1316 (19.6%)	1442 (20.1%)	1532 (20.1%)	5872 (19.9%)
	> = 30	934 (11.6%)	814 (12.1%)	801 (11.2%)	933 (12.3%)	3482 (11.8%)
	Missing	262 (3.3%)	205 (3.0%)	228 (3.2%)	287 (3.8%)	982 (3.3%)
CD4 cell count, cells/mm3	<50	488 (6.1%)	467 (6.9%)	460 (6.4%)	330 (4.3%)	1745 (5.9%)
	50–199	627 (7.8%)	688 (10.2%)	710 (9.9%)	681 (9.0%)	2706 (9.2%)
	> = 200	1404 (17.5%)	1444 (21.5%)	1472 (20.5%)	1444 (19.0%)	5764 (19.5%)
	Missing	5509 (68.6%)	4128 (61.4%)	4529 (63.2%)	5150 (67.7%)	19,316 (65.4%)
CD4 percentage, %	<25	63 (0.8%)	21 (0.3%)	0 (0.0%)	0 (0.0%)	84 (0.3%)
	> = 25	20 (0.2%)	4 (0.1%)	0 (0.0%)	0 (0.0%)	24 (0.1%)
	Missing	7945 (99.0%)	6702 (99.6%)	7171 (100.0%)	7605 (100.0%)	29,423 (99.6%)
Measurement of CD4 count or percent	Have at least one	2577 (32.1%)	2619 (38.9%)	2642 (36.8%)	2455 (32.3%)	10,293 (34.9%)
	Missing both	5451 (67.9%)	4108 (61.1%)	4529 (63.2%)	5150 (67.7%)	19,238 (65.1%)
Facility type	Dispensary	815 (10.2%)	929 (13.8%)	1206 (16.8%)	1477 (19.4%)	4427 (15.0%)
	Health centre	2395 (29.8%)	2129 (31.6%)	2418 (33.7%)	2750 (36.2%)	9692 (32.8%)
	Hospital	4366 (54.4%)	3354 (49.9%)	3297 (46.0%)	3155 (41.5%)	14,172 (48.0%)
	Other	437 (5.4%)	301 (4.5%)	224 (3.1%)	199 (2.6%)	1161 (3.9%)
	Missing	15 (0.2%)	14 (0.2%)	26 (0.4%)	24 (0.3%)	79 (0.3%)

Of the 29,531 children enrolled in care, a total of 10,390 (35%) were in WHO stage III and 3196 (11%) were in WHO stage IV (Table 1). The proportion of children with CD4 count <50 cells/mm3 decreased in 2014 compared to earlier years (4.3% in 2014 versus 5.9% overall). However, approximately two-thirds of children across all years were missing a baseline CD4 absolute count. The proportion of children missing CD4 percentage data was even higher, at nearly 100% overall. Only 35% of children had either a CD4 count or percentage recorded.

The most common referral point was voluntary counselling and testing (VCT) (18% overall), followed by provider initiated testing and counselling (PITC) (12%). However, 65% of children did not have their referral point recorded (Table 2).

**Table 2:** Referral sources, prophylaxis and nutritional status of 29,531 children enrolled into care between 2011 and 2014

Referral source		2011	2012	2013	2014	Total
	HBC	48 (0.6%)	28 (0.4%)	38 (0.5%)	23 (0.3%)	137 (0.5%)
	PITC	894 (11.1%)	748 (11.1%)	934 (13.0%)	1040 (13.7%)	3616 (12.2%)
	PMTCT	330 (4.1%)	310 (4.6%)	285 (4.0%)	364 (4.8%)	1289 (4.4%)
	VCT	1524 (19.0%)	1329 (19.8%)	1287 (17.9%)	1284 (16.9%)	5424 (18.4%)
	Missing	5232 (65.2%)	4312 (64.1%)	4627 (64.5%)	4894 (64.4%)	19,065 (64.6%)
Positive for TB	No	27,658 (93.7%)	7359 (91.7%)	6251 (92.9%)	6780 (94.5%)	7268 (95.6%)
	Yes	672 (2.3%)	117 (1.5%)	149 (2.2%)	211 (2.9%)	195 (2.6%)
	Missing	1201 (4.1%)	552 (6.9%)	327 (4.9%)	180 (2.5%)	142 (1.9%)
Cotrimoxazole	No	7977 (27.0%)	2264 (28.2%)	2026 (30.1%)	1835 (25.6%)	1852 (24.4%)
	Yes	21,554 (73.0%)	5764 (71.8%)	4701 (69.9%)	5336 (74.4%)	5753 (75.6%)
Malnourished	No	19,172 (64.9%)	3370 (42.0%)	4540 (67.5%)	5272 (73.5%)	5990 (78.8%)
	Mild	3 (0.0%)	3 (0.0%)	0 (0.0%)	0 (0.0%)	0 (0.0%)
	Moderate	3005 (10.2%)	453 (5.6%)	806 (12.0%)	911 (12.7%)	835 (11.0%)
	Severe	906 (3.1%)	158 (2.0%)	268 (4.0%)	259 (3.6%)	221 (2.9%)
	Missing	6445 (21.8%)	4044 (50.4%)	1113 (16.5%)	729 (10.2%)	559 (7.4%)

Approximately 2–3% of children were identified at enrolment as being positive for TB and this was fairly constant across the years (Table 2). TB screening was done using a standardized TB screening tool (annex). All children <5 years old are given cotrimoxazole and for >5 years with CD4 cell count below 350. The proportion of children on cotrimoxazole over the years has also remained fairly constant, at 70–74%. In earlier years, the nutritional status of children at enrolment was not consistently captured (22% missing in 2011 and 50% missing in 2012). In 2014, the missing data was a lot lower (10%). Across all years, the majority of children were not malnourished, with only 2–4% recorded as being severe malnourished (Table 2).

### Eligibility at enrolment and time to starting ART

Among the 21,503 children enrolled in 2012–2014, 15,522 (72%) were eligible for ART from enrolment (Table 3). However, 4624 (22%) had unknown eligibility, mainly due to either being categorized as WHO stage I or II or missing CD4 count or percentage. The proportion with unknown eligibility was slightly lower among older compared to younger children except for <2 year olds, who were all eligible for treatment initiation regardless of WHO staging or CD4 results.

**Table 3:** Eligibility for ART initiation by age at enrolment

Age category II, years	<2	2–5	6–9	10–14	Total
Eligible	6073 (100%)	3840 (61%)	2855 (60%)	2754 (63%)	15,522 (72%)
Not eligible	0	317 (5%)	603 (13%)	437 (10%)	1327 (6%)
Unknown eligibility	0	2088 (33%)	1326 (28%)	1210 (27%)	4624 (22%)
Total	6073 (100%)	6245 (100%)	4784 (100%)	4401 (100%)	21,503 (100%)

Among <2 year olds, who should all start treatment immediately regardless of WHO stage or CD4 results, 41% initiated treatment on the day of enrolment into CTC care, and a further 24% initiated within 1 month of enrolment (Table 4). However, around 7% started treatment >3 months after enrolment, and 21% had not started treatment at all.

**Table 4:** Time from enrolment to ART initiation by age

	<2	2–5	6–9	10–14	Total
Enrolment	2468 (40.6%)	1398 (22.4%)	874 (18.3%)	753 (17.1%)	5493 (25.5%)
Within 1 month	1439 (23.7%)	1583 (25.3%)	1318 (27.6%)	1468 (33.4%)	5808 (27.0%)
Within 2 months	316 (5.2%)	426 (6.8%)	346 (7.2%)	330 (7.5%)	1418 (6.6%)
Within 3 months	150 (2.5%)	185 (3.0%)	148 (3.1%)	172 (3.9%)	655 (3.0%)
Within 6 months	196 (3.2%)	288 (4.6%)	270 (5.6%)	200 (4.5%)	954 (4.4%)
Within 12 months	142 (2.3%)	275 (4.4%)	239 (5.0%)	195 (4.4%)	851 (4.0%)
>12 months	90 (1.5%)	220 (3.5%)	179 (3.7%)	137 (3.1%)	626 (2.9%)
Not yet started	1272 (20.9%)	1870 (29.9%)	1410 (29.5%)	1146 (26.0%)	5698 (26.5%)
Total	6073 (100%)	6245 (100%)	4784 (100%)	4401 (100%)	21,503 (100%)

### ART initiation by age

A total of 15,805 children were observed to initiate ART by the end of December 2014 (Table 4). The proportion by age were 13% aged <1 year, 36.2% aged 1–4 years, 26.6% aged 5–9 years, 20.6% aged 10–14 years and <1% aged 14 + years (Table 5).

**Table 5:** ART initiation by age

		2012	2013	2014	Total
	Age	3876 (24.5%)	5488 (34.7%)	6441 (40.8%)	15,805 (100%)
Age category I	<1	631 (16.3%)	681 (12.4%)	745 (11.6%)	2057 (13.0%)
	1–4	1403 (36.2%)	2065 (37.6%)	2506 (38.9%)	5974 (37.8%)
	5–9	1031 (26.6%)	1562 (28.5%)	1764 (27.4%)	4357 (27.6%)
	10–14	797 (20.6%)	1146 (20.9%)	1363 (21.2%)	3306 (20.9%)
	>14^a^	14 (0.4%)	34 (0.6%)	63 (1.0%)	111 (0.7%)

^a^ Captured aged <15 years at enrolment but became >14 years old before starting treatment

### Nutritional status

**Fig. 1:**
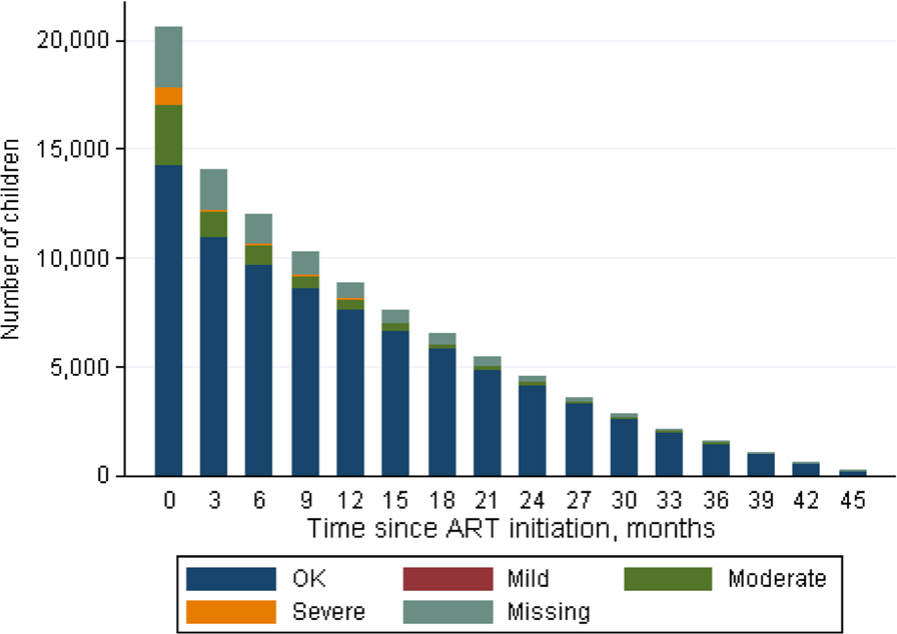
Nutritional status of children over time from ART initiation

Figure 1 shows the nutritional status of children over time from ART initiation. The declining bars show that fewer children are being followed up for long after ART initiation. Overall most children were not malnourished, and there was little severe malnutrition observed after 3 months on ART.

### Mortality of children

**Fig. 2:**
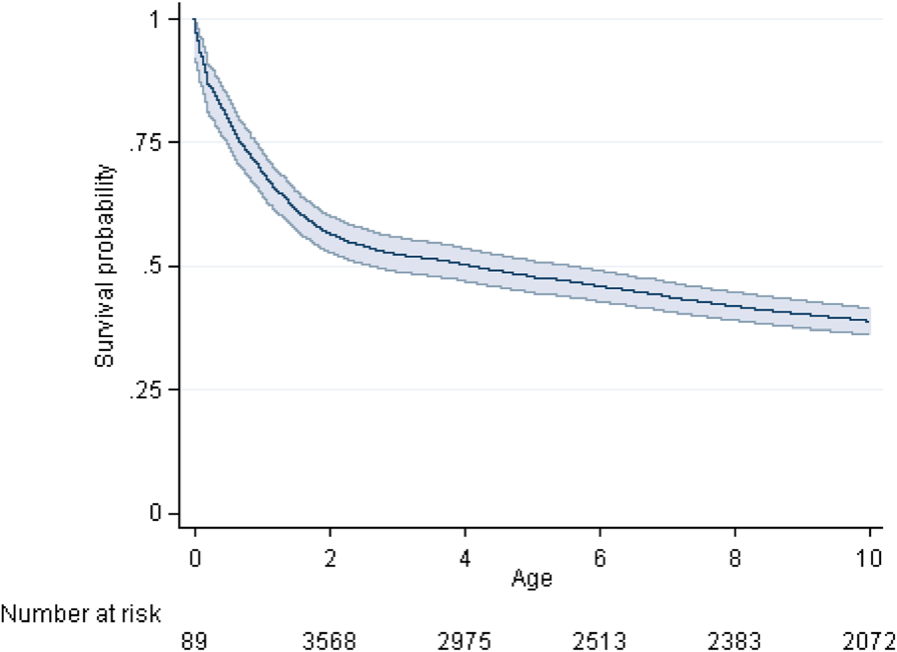
Survival of HIV positive children from birth (age in years)

**Fig. 3:**
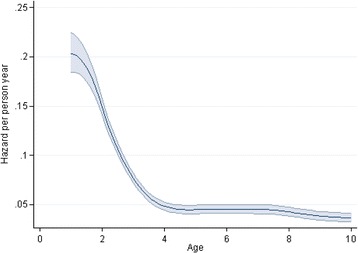
Hazard from birth in HIV positive children (age in years)

Among 29,531 children enrolled in 2011–2014, 2368 (8%) were known to have died, 9243 (31%) were considered LTFU and 17,920 (61%) were still under active follow up. Fig. 2 shows the survival probability over age. The probability of death by 1 year of age was 31% (95% CI 26–35), by 2 years of age was 43% (40–47), by 5 years of age was 52% (49–55) and by 10 years of age was 61% (58–64). The hazard of death was greatest at very young ages (<2 years old), decreased sharply by 4 years old and then plateaued, with relatively constant hazard from 4 to 10 years old (Fig. 3).

**Fig. 4:**
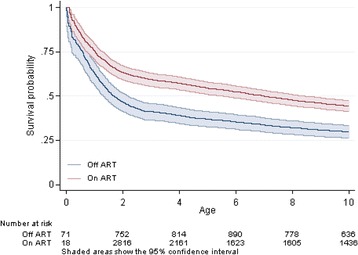
Survival from birth, in HIV positive children, by ART status

**Fig. 5:**
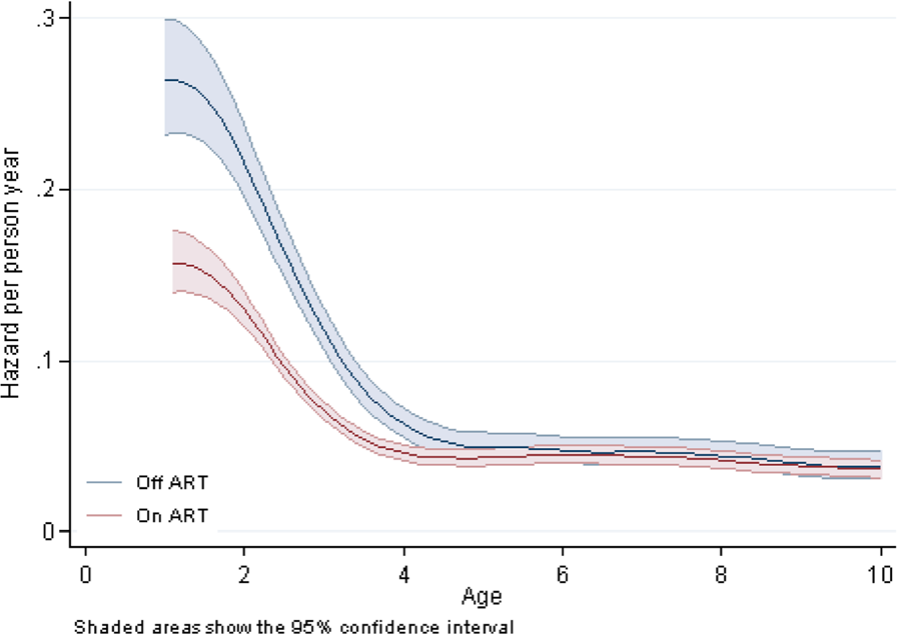
Hazard from birth, in HIV positive children, by ART status

Children who were on ART had around 10–15% higher survival over time (Fig. 4). The hazard was substantially higher among younger chilren not on ART compared to those on ART (Fig. 5). However, from 4 years of age onwards, there was little difference in the hazards between children irrespective of ART status.

## Discussion

Our results show that the number of children enrolled into HIV care and treatment programme per year is between 6700 and 8000 and that the proportion of girls is slightly higher compared to boys. Almost 72% of the enrolled children were eligible for ART at the time of enrolment, and that almost half (46%) were in WHO stages III and IV, indicative of advanced disease stage. Mortality rate among the enrolees was about 8%, and the hazard was substantially higher among younger children not on ART compared to those on ART.

Provision of ART as soon as the child is diagnosed is the biggest factor in improving survival, with a hazard of mortality in the first year of life, at 15% for children on ART, compared to 26% for children not on ART. Children who were on ART had around 10–15% higher survival over time. However, from 4 years of age onwards, there was little difference in the hazards between children irrespective of ART status. It is likely that children who reach older ages without having started treatment are likely to be be “long term non-progressors”, refering to individuals who are infected with HIV, but maintain a CD4 count greater than 500 without antiretroviral therapy with a detectable viral load [14].

The benefits of ART observed in this study have been demonstrated in several studies showing significant reduction mortality with early ART initiation, within the first 12 weeks of life [15–23], accompanied with a greater than 90% probability of survival into adulthood [24].

Our study revealed a number of significant challenges that need considerable attention by National AIDS Control Programme and other stakeholders involved in the provision of paediatric HIV care and treatment services in Tanzania. We noted that i) most children have advanced disease at the time of enrolment ii) approximately two-thirds of children were missing a baseline CD4 measurement and only 35% of children had either a CD4 count or percentage recorded, indicating limited access to CD4 testing services, and iii) 31% were lost to follow-up (LTFU).

It is therefore a critical public health priority to address these challenges for a successful expansion of paediatric HIV care and treatment services in Tanzania. Strategies for early diagnosis of HIV-infected children and linking them to care and treatment services are essential. Besides this, minimizing the current attrition rates, which have also been observed in clinical settings across all HIV programs in Africa, with overall attrition rates ranging from 10% to more than 50% at 24 months [25–30], mainly because of loss to follow-up (LTFU) or AIDS-related death [27, 31]. One possible explanation of the high rates of LTFU could associated with the quality of the data collection and reporting systems, which is a common challenge of health information systems in resource-limited settings [32–34]. Improving capacity for systematic monitoring and accurate reporting may reduce the number of patients LTFU, particularly for sites that rely on paper-based monitoring systems.

Despite these challenges it was encouraging to note that majority of children had good nutrition status, with only 2–4% of children severely malnourished and that nutritional status improved with time on ART. Furthermore, only 2–3% of children were diagnosed with TB at the time of enrolment and that the proportion of children on Cotrimoxazole was high (70–74%).

### Limitations of the study

This report has analysed a large amount of data from across Tanzania, but there are several limitations to our findings. One limitation is that the data come from the electronic, CTC database, which is used in the larger and better run clinics. The situation reported here might not be the same in smaller health facilities that have not provided electronic data. Secondly the data are dependent on the self-reporting of conditions (such as opportunistic infections) by patients, and the recording of such data by clinicians and nurses. The amount of missing data has been reduced over the past 5 years but it is still a problem.

We have used basic statistical methods to analyse these data. Some of the assumptions around independence may not be valid in the analysis of these data, especially in the treatment of missing data (which is likely to be correlated with the outcomes). We have used a competing risks analysis of the mortality pre-ART, which is a first step in acknowledging these concerns about the use of basic statistical methods. However further work needs to be done about the theoretical underpinnings of the statistical methods used for the analysis of routinely collected data.

Despite its limitations, this review has provided some insights in to the achievements and gaps in clinical services for paediatric HIV care and treatment in Tanzania and does suggest areas that could be strengthened to improve these services.

## Conclusions

Significant progress has been made regarding provision of paediatric HIV care and treatment in Tanzania. On average 7000 children are enrolled annually, and that approximately two thirds of children diagnosed under the age of 2 years were initiated on ART within a month. Provision of ART as soon as the child is diagnosed is the biggest factor in improving survival, with a hazard of mortality in the first year of life, at 15% for children on ART, compared to 26% for children not on ART. However we noted that i) most children had advanced disease at the time of enrolment ii) approximately two-thirds of children were missing a baseline CD4 measurement and only 35% of children had either a CD4 count or percentage recorded, indicating limited access to CD4 testing services, and iii) 31% were lost to follow-up (LTFU). These challenges underscore the need for more aggressive strategies to increase enrolment and retention of children in such settings. Evidently more work is needed to link HIV-infected children to HIV services.

## Abbreviations

ART: Anti-retroviral Therapy
CD4: Cluster of Differentiation 4
CDC: Centres for Disease Control
CTC: Care and Treatment Clinic
HIV: Human Immunodeficiency Virus
LTFU: Lost To Follow Up
NACP: National AIDS Control Programme
NIMR: National Institute for Medical Research
NLOT: No Longer On Treatment
UNAIDS: United Nations Programme on HIV/AIDS
WHO: World Health Organization
